# Two-Stage Exchange Arthroplasty for Periprosthetic Reverse Shoulder Arthroplasty Infection Provides Comparable Functional Outcomes to Primary Reverse Shoulder Arthroplasty

**DOI:** 10.3390/jcm13030904

**Published:** 2024-02-04

**Authors:** Maristella Francesca Saccomanno, Alexandre Lädermann, Philippe Collin

**Affiliations:** 1 Department of Medical and Surgical Specialties, Radiological Sciences and Public Health, University of Brescia, 25123 Brescia, Italy; maristellasaccomanno@hotmail.it; 2 Department of Bone and Joint Surgery, Spedali Civili, 25123 Brescia, Italy; 3 Division of Orthopaedics and Trauma Surgery, Hôpital de La Tour, Rue J.-D. Maillard 3, 1217 Meyrin, Switzerland; 4 Division of Orthopaedics and Trauma Surgery, Department of Surgery, Geneva University Hospitals, Rue Gabrielle-Perret-Gentil 4, 1205 Geneva, Switzerland; 5 Faculty of Medicine, University of Geneva, Rue Michel-Servet 1, 1211 Geneva, Switzerland; 6 CHP Saint-Gregoire, 6 Boulevard de la Boutière, 35760 Saint-Grégoire, France; docphcollin@gmail.com; 7 Clinique Victor Hugo, 5 Bis Rue du Dôme, 75016 Paris, France; 8 American Hospital of Paris, 55 Boulevard du Château, 92200 Neuilly-sur-Seine, France

**Keywords:** PJI, reverse prosthesis, revision, complication, PROMs, results

## Abstract

This study aimed to compare functional outcomes after two-stage revision reverse shoulder arthroplasty (RSA) for periprosthetic joint infection (PJI) with the results of primary RSA. Patients affected by PJI and treated by means of two-stage revision RSA were 1:1 matched with a group of patients who were treated electively with RSA without developing any complications. Out of 1477 RSAs performed between 2009 and 2021, 16 patients developed a PJI. Each matched cohort comprised 16 patients (3 females, 13 males). The mean age was 69.13 ± 5.43 years old in the PJI group and 70.28 ± 5.04 (*p* = 0.543) in the matched cohort. The mean follow-up was 41.23 ± 26.9 months in the PJI group and 28.5 ± 20.2 (*p* = 0.142) in the matched group. Only one patient showed recurrent PJI five years after revision RSA. Comparison between the PJI patients and matched patients did not show any significant differences at the latest follow-up, nor for subjective shoulder value (SSV) (*p* = 0.101) or Constant score (*p* = 0.134). Two-stage exchange RSA for PJI allows for appropriate control of the disease and good functional outcomes. Comparison with an age- and sex-matched cohort of uninfected patients showed no significant differences, thus confirming the idea that revision surgeries may lead to satisfactory functional outcomes, as expected after primary surgery.

## 1. Introduction

Periprosthetic shoulder infections (PJIs) represent a diagnostic and therapeutic challenge [1]. The rate of PJI after primary shoulder arthroplasty is reported to be as high as 6% during the first two postoperative years [2,3,4]. 

Surgical management can range from debridement, antibiotics, and implant retention (DAIR) [5] to single or multi-staged revision to resection arthroplasty or even amputation in worst-case scenarios. DAIR is usually the most common option in the case of acute infection [5]. The superiority of a multi-stage procedure over a one-stage revision has been controversially discussed in the literature without reaching a definitive conclusion [6,7,8,9]. However, two-stage revision remains the standard of care in the chronic setting. Few case series have reported acceptable functional outcomes after revision arthroplasty in the setting of PJIs, but most of the time, functional evaluation before implanting the primary replacement has not been reported [2,3,10,11,12].

Thus, the aim of the present paper was to determine functional outcomes after two-stage exchange procedures for PJIs after reverse shoulder arthroplasties (RSAs). The hypothesis of the study was that a two-stage revision does not affect the final functional outcome. 

## 2. Materials and Methods

### 2.1. Patient Selection

A retrospective study was conducted on prospectively collected data. All patients treated with a two-stage revision protocol for a shoulder arthroplasty infection from 2009 to 2021 with a minimum of one year of clinical follow-up were included. The inclusion criteria consisted of all consecutive adult patients who underwent a two-stage revision RSA previously implanted for primary osteoarthritis, cuff tear arthropathy, fractures, or fracture sequelae. The exclusion criteria were patients with a history of native septic shoulder arthritis, periprosthetic fractures, or neurological diseases or immunocompromised patients. The cohort was then 1:1 matched for age and sex with a group of patients treated electively with shoulder replacement without developing any complication in the same time frame. The surgical approach and type of implant were the same in the two cohorts, as the surgeon was the senior author in all cases. The identified patients’ electronic medical records were reviewed, so the patients’ demographics, comorbidities, preoperative diagnosis, preoperative treatments, surgical details, time to revision, and microbiology data were collected. The present study was approved by the Institutional Review Board (CERC-VS-2023-03-4). All participants gave written informed consent before data collection began. The study was conducted according to the principles of good clinical practice and those of the Declaration of Helsinki and its updated version (Tokyo, Japan, 2004).

### 2.2. Infection Definition and Postoperative Assessment

PJIs were diagnosed in accordance with the definition given by the International Consensus Meeting on Orthopedic Infection held in 2018 [13], which focused on the shoulder. Therefore, based on this, the major criteria were:

- Presence of a sinus tract from the skin surface to the prosthesis;
- Gross intra-articular pus;
- Two positive tissue cultures with phenotypically identical virulent organisms.

Otherwise, minor criteria were applied, taking into consideration: tissue biopsies, radiological loosening of the humeral components, and laboratory markers (white blood cell counts, C-reactive protein, and erythrocyte sedimentation rate) [13,14]. Probable and possible PJIs were defined according to the score proposed by the Consensus meeting [13] and treated using DAIR in the case of acute PJIs or two-stage revisions in subacute and chronic cases.

PJIs were categorized as acute if they occurred within three months after the index procedure, subacute between 3 and 12 months, or chronic if they occurred after this period [15]. In the case of an acute PJI, a DAIR approach was first attempted [5]. All DAIRs were open-surgery procedures. The previous surgical approach was followed with excision of the sinus tract, if any. Samples of the synovial fluid and the affected tissues were sent to the laboratory for microbiological analysis, and, in some cases, the explanted modular components were sonicated. Radical debridement of all nonviable tissues was then performed, followed by profuse irrigation with at least 9 L of saline. A suction drain was left in situ until there was minimal output. Tailored antibiotics, as recommended by antimicrobial stewardship, were given after surgery for at least six weeks. A large spectrum empirical antibiotic was used if cultures yielded no microbial isolation [16]. 

If the procedure failed, a two-staged revision was then performed. The failure of the DAIR procedure was defined as follows: (1) the presence of local or systemic infectious symptoms; (2) laboratory signs suggesting the presence of a PJI; and (3) signs of loosening on radiography.

Patients directly affected by subacute or chronic infections underwent a two-stage revision.

In the interval between implant removal and reimplantation, patients underwent laboratory tests (white blood cell counts, C-reactive protein, and erythrocyte sedimentation rate) and local inspection of the wound monthly.

### 2.3. Two-Stage Revision Arthroplasty Protocol

Revision surgeries were always performed by the senior author (P.C.), and all patients were treated using the same protocol. A deltopectoral approach was used in all cases. All hardware and sutures, as well as polymethyl methacrylate (PMMA), if any, were removed, followed by extension soft-tissue and bone irrigation and debridement. At least five tissue samples were collected for culture evaluation. An antibiotic-loaded cement spacer was handmade for each patient by using the smallest humeral stem available (diameter = 6.5; length = 100 mm; Aequalis^®^ Shoulder System, Stryker, Kalamazoo, MI, USA) covered by gentamicin-impregnated PMMA (Palacos R+G; Heraeus Medical, Wehrheim, Germany). Care was taken to not reinsert the subscapularis tendon if not possible. Tailored antibiotics, as recommended by antimicrobial stewardship, were given for at least six weeks. A large spectrum empirical antibiotic was used if cultures yielded no microbial isolation [16]. 

Second-stage reimplantation was scheduled after the completion of antibiotic therapy based on wound status and the course of blood infection parameters. The type of prosthesis used during the reimplantation was the Aequalis Reverse (Aequalis^®^ Shoulder System, Stryker) in all cases.

### 2.4. Postoperative Rehabilitation

The arm was immobilized in a sling with an abduction pillow for four weeks. The rehabilitation process followed a previously published protocol [17]: passive forward flexion and external rotation were allowed immediately, whereas active range of motion started after sling removal. Activities of daily living were allowed, but strengthening was not specifically recommended.

### 2.5. Functional Outcomes

All patients underwent clinical and radiological examination at standardized endpoints set before primary replacement and after reimplantation at one, three, and six months and then yearly thereafter. From a clinical standpoint, two scores were used: the subjective shoulder value (SSV) [18] and the Constant score [19]. The SSV provides a subjective evaluation of shoulder function, expressed as a percentage of a normal shoulder. This score ranges from 0 to 100%. The Constant score is an instrument to objectively evaluate overall shoulder function. It is a 100-point scale composed of four subscales: pain (15 points), activities of daily living (20 points), strength (25 points), and range of motion: forward elevation, external rotation, abduction, and internal rotation of the shoulder (40 points). The higher the score, the higher the quality of the function. 

### 2.6. Radiological Analysis

Standardized radiographs in anteroposterior in neutral, external and internal rotations, and axillary lateral views were obtained preoperatively and postoperatively to observe signs of loosening, osteolysis, and modifications in implant position.

### 2.7. Statistical Analysis

Statistical analysis was performed using GraphPad (version 9, GraphPad Software, San Diego, CA, USA). Data were expressed as frequencies (percentages) for categorical variables and means and standard deviations (SDs) for continuous variables as verified for normal distribution using the Shapiro–Wilk test. A paired *t*-test was performed to compare preoperative with latest follow-up status. An unpaired *t*-test was performed to compare the two cohorts of PJI with matched patients. Significance was defined as *p* < 0.05.

## 3. Results

Out of 1477 shoulder replacements performed between 2009 and 2021, 16 (1%) patients developed a PJI. Therefore, each matched cohort comprised 16 patients (3 females, 13 males). The mean age at the time of index surgery was 69.13 ± 5.43 years old in the PJI group (group A) and 70.28 ± 5.04 (*p* = 0.543) in the matched cohort (group B). The two cohorts were homogenous for age, sex, preoperative diagnosis, and comorbidities. The patients’ characteristics are summarized in Table 1.

All patients who underwent primary shoulder replacement for primary osteoarthritis or rotator cuff arthropathy had previously been treated conservatively with physiotherapy for at least six months. On the contrary, some patients with fracture sequelae had undergone previous surgical treatment. In group A, one fracture was previously treated with a nail, while the other was treated conservatively, but the patient previously underwent a Latarjet procedure. In group B, two patients out of three underwent previous surgery. One fracture was treated surgically with a nail, while the two others were treated conservatively, but one of the patients previously underwent a Latarjet procedure. Physical therapy was attempted in all patients. Pain and stiffness were the main indications for shoulder replacement in patients affected by fracture sequelae.

Primary implants were all uncemented.

In group A, three patients were not included in the clinical evaluation at the last follow-up: two patients kept the spacer long-term, while another died before reimplantation. One of the two patients who decided to keep the spacer refused clinical evaluation, while the other showed an SSV = 30 and a Constant score = 29. No other patients were lost at follow-up. The mean time between implant removal and reimplantation was 4.36 ± 1.2 months. The mean follow-up was 41.23 ± 26.9 months in the PJI group (group A) and 28.5 ± 20.2 (*p* = 0.142) in the matched group (group B). An early PJI was diagnosed in three patients; therefore, a DAIR procedure was first attempted, but it failed, so all patients underwent a two-stage revision. Five patients affected by subacute PJI underwent implant removal at a mean of 6.2 + 2 months from the index procedure, while the remaining patients with chronic PJI underwent implant removal at a mean of 15.1 + 1.5 months from the index procedure. Cutibacterium acnes were isolated in 11 cases out of 16 (68%). Negative tissue cultures were found in two patients, but a major criterion (sinus tract) was present at the diagnosis, so they were considered infected. 

All patients showed at least one of the major criteria [13].

Isolated microorganisms and antibiotics are reported in Table 2. The two patients with negative cultures were treated with vancomicine for eight weeks. Tailored antibiotics were administered in the case of positive cultures. The mean administration of antibiotic therapy was 12.9 weeks (range 6–24 weeks)

At last follow-ups, all patients but one showed significant functional improvement (Table 3). 

All patients showed no radiographic signs of recurrent PJI (i.e., component loosening). Notably, only one reinfection was detected five years after the revision RSA. This patient was the only one who did not show significant improvement in SSV and Constant score. No other complications were reported.

In group B, all patients showed significant functional improvement (Table 4).

Comparison between the PJI group (group A) and the matched patients (group B) did not show any significant differences between and within groups at one year and at the latest follow-up either for SSV (*p* = 0.101) (Table 5) or for Constant score (*p* = 0.134) (Table 6). 

A subgroup analysis in group A showed no differences at final follow-up between patients who underwent DAIR and patients who did not (Table 7).

## 4. Discussion

The main findings of the present study were the low prevalence of PJIs, the successful two-stage exchange arthroplasty for PJIs allowing appropriate control of the disease, and good functional outcomes, confirming our hypothesis. All patients showed better patient-reported outcome measures compared to the situation before primary implant and no differences compared to an age- and sex-matched cohort.

Of note, 81% of patients (13 out of 16) were male. It has been shown that men have a higher burden of Cutibacterium acnes around the shoulder compared with their female counterparts [20]. The bacterial spectrum was comparable to that reported in the previous literature, with Cutibacterium acnes being the predominant pathogen, followed by Staphilococci [21]. Less common compared to the literature was the finding of multiple germ isolations in two acute and chronic cases. The reinfection rate in this study was 6% (1/16 patients), comparable to the literature [7]. The only patient who experienced a reinfection was an 80-year-old man with a pacemaker. The isolated microorganism was Cutibacterium acnes. It is probable that overall health condition may have somehow contributed to reinfection.

In the present series, DAIR appears to be ineffective in acute cases. Of note, functional outcomes at the latest follow-up were not affected by the DAIR procedure. However, having three cases only does not allow for a definitive conclusion. Looking deeper into the current literature results, the topic is still the subject of ongoing discussion, since the latest review reported a wide range of PJI control rates, between 11.1% and 100% [5]. However, the efficacy of DAIR was surely beyond the purpose of the study. On the other hand, the PJI control rate after two-stage revisions was 94% (15 cases out of 16) in the present study, while the literature results vary between 63% and 100% [16]. Such high variability in the literature results can probably be explained by the following. Despite the abundance of studies focusing on PJIs, there is widespread heterogeneity in definition, diagnosis, and treatment protocols. This is mainly because in the absence of definitive guidelines, lessons learned from the hip and knee have been historically adapted to the shoulder. However, given the lack of a reliable and consistent definition, the treatment approaches have been contrasting. In 2019, Garrigues et al. [13,14,16,22] published the results of a consensus meeting held in 2018 focusing on four main topics: definition, evaluation, prevention, and management of PJIs. Although consensus meetings do not provide guidelines, they can at least be considered a starting point, so future studies can rely on their assumptions to speak a common language. Therefore, the current study relied on the consensus results to better define the infectious status.

Focusing on treatment strategies, studies reporting results after two-stage revisions for PJIs are mainly retrospective case series [2,3,10,11,12]. One of the issues around the debate between single- and two-stage revision is the risk of increasing soft tissue damage and bone loss with multiple surgeries. 

Therefore, the main goal of studies on two-stage revisions has always been to provide information about functional recovery in addition to PJI control. Tseng et al. [12] compared the functional outcomes of 28 multi-stage revision arthroplasties for PJIs to 27 aseptic revision RSAs. The authors showed no differences in range of motion, pain visual analog scale (VAS), and American Shoulder and Elbow Surgeons (ASES) subjective score between groups, thus remarking that revision procedures can yield good results. In the present study, the risk of bone loss was not specifically investigated, but the PJI cohort was compared to patients who underwent a primary RSA with no further complications, thus highlighting that functional results could be as successful as expected in a primary setting, even in the case of PJI and staged revisions. Two studies [23,24] compared outcomes in patients who underwent two-stage revisions and patients who kept the antibiotic spacer as definitive management. Both treatments provided PJI eradication and satisfying subjective results. On the other hand, two-stage revisions generated obviously superior range of motion and functional outcome scores. The mean Constant score in the two-stage revision groups was lower than the results reported in the present study. However, it must be considered that follow-up was much longer in the other two studies [23,24]. Moreover, Pellegrini et al. [24] also reported pre- and postoperative changes in functional outcomes without making any distinction between the two groups of patients. 

In the present study, the authors preferred not to include patients who kept the spacer in the last clinical evaluation. Antibiotic spacers do not aim to restore function. Patients who decided to maintain it as definitive management preferred to avoid another surgery.

Kim et al. [10] recently reported functional outcomes after two-stage RSA following a temporary antibiotic-loaded cement spacer for shoulder infection in 11 patients at a mean follow-up of 29.9 months (range 12–48). The authors included different baseline conditions: extensive osteomyelitis with joint destruction, osteomyelitis with advanced degenerative osteoarthritis, massive rotator cuff tear, and PJIs. They reported improved patient-reported outcome measures (PROMs) after RSA overall. In particular, the SSV results at the final follow-up were comparable to the results of the present study.

Understandably, the retrospective nature of these studies does not provide preoperative information in most cases. Moreover, assuming that infections are strictly related to clinical failure, studies are unlikely to compare PROMs before implant removal to after revision. 

However, it is the author’s opinion that what matters for patients is ultimately to improve function and solve pain issues as opposed to the length of the reconstruction process. In the future, it could be interesting to assess functional outcomes more objectively through in vivo biomechanical analysis [25].

### Strengths and Limitations

To the best of our knowledge, the present study is the first to prove that revision arthroplasties can lead to successful functional recovery, thus improving overall shoulder function not only when compared to before the index procedure but also when compared with patients who have undergone the same primary surgery without complications. The main strength of the present study is that this assumption has been proven subjectively through the SSV score and objectively through the Constant score. Moreover, Hao et al. [26] recently established that the minimal clinical important difference for Constant score after aseptic revision is 12.6 points. In the present study, the PJI group showed an improvement of 24 points. There are also some limitations. Firstly, this study was retrospective in design, regardless of prospective data collection. Secondly, and despite a consequent cohort, the number of finally included patients was undoubtedly small, especially for subgroup analysis, although comparable with previously reported series [3,10,11,12], confirming the efficacy and safety of RSA. Moreover, a preoperative aspirate was never performed. Although the arthrocentesis represents a mandatory step in the workup of knee and hip periprosthetic infection, its role in shoulder arthroplasty remains controversial [14]. Several authors have reported a notable occurrence of “dry taps” (up to 44%) even in cases where infection was confirmed during revision arthroplasty [27]. Furthermore, when fluid is successfully obtained, apprehension exists regarding the sensitivity and specificity of currently available tests, which may not be sufficient to consistently rule out an infection [28].

## 5. Conclusions

Two-stage exchange arthroplasty for PJIs allows for appropriate control of the disease and good functional outcomes. Comparison with an age- and sex-matched cohort of uninfected patients showed no significant differences, thus confirming the idea that revision surgeries may lead to satisfactory functional outcomes, as expected after primary surgery. In the present study, DAIR showed low chance of success in cases of acute PJI.

## Figures and Tables

**Table 1:** Baseline characteristics: group A (PJI patients) and B (matched group).

Variable	Group A (*n* = 16)	Group B (*n* = 16)	*p*
Age	69.13 ± 5.43	70.28 ± 5.04	0.54
Sex (M/F)	13/3	13/3	-
Diagnosis	CTA = 9 OA = 5 Fracture sequelae = 2	CTA = 9 OA = 4 Fracture sequelae = 3	-
Comorbidities	Hypercholesterolemia: 3 Heart disease: 1		
Primary procedure	RSA	RSA	-
Baseline SSV	45.44 ± 18.08	35.63 ± 13.28	0.14
Baseline Constant score	42.56 ± 22.71	43.75 ± 18.18	0.87
Mean follow-up (months)	41.23 ± 26.9	28.5 ± 20.2	0.14

CTA: cuff tear arthropathy; OA: osteoarthritis; RSA: reverse shoulder arthroplasty; SSV: shoulder subjective value.

**Table 2:** Microorganism isolation.

Patient Number	Type of Infection	Microorganism	Antibiotic Therapy
1	Acute	Cutibacterium acnes	Clindamycin
2	Acute	Cutibacterium acnes/ Staphilococcus Epidermidis	Clindamycin and rifampicin
3	Subacute	Cutibacterium acnes	Linezolid
4	Subacute	Cutibacterium acnes/ Staphilococcus Epidermidis	Clindamycin
5	Chronic	Cutibacterium acnes	Amoxicillin + clavulanic acid
6	Chronic	Cutibacterium acnes	Amoxicillin + clavulanic acid
7	Subacute	Cutibacterium acnes	Amoxicillin + clavulanic acid
8	Acute	Cutibacterium acnes	Amoxicillin + clavulanic acid
9	Subacute	Streptococcus mitis	Clindamycin and rifampicin
10	Chronic	Cutibacterium acnes	Clindamycin and rifampicin
11	Chronic	Cutibacterium acnes	Amoxicillin + clavulanic acid
12	Chronic	Cutibacterium acnes	Clindamycin
13	Chronic	negative	Vancomicine
14	Chronic	negative	Vancomicine
15	Chronic	Staphilococcus Epidermidis	Clindamycin
16	Subacute	Cutibacterium acnes	Clindamycin

**Table 3:** Comparison between baseline and follow-up conditions for functional outcomes in group A (PJI patients).

Outcome	Baseline (Mean ± SD)	Follow-Up (Mean ± SD)	*p*-Value
SSV	45.44 ± 18.08	72.50 ± 13.89	0.02
Constant score	42.56 ± 22.71	66.85 ± 15.99	0.001

SSV: shoulder subjective value.

**Table 4:** Comparison of functional outcomes between baseline and last follow-up in group B.

Outcome	Baseline (Mean ± SD)	Follow-Up (Mean ± SD)	*p*-Value
SSV	35.63 ± 13.28	82.50 ± 8.86	0.0001
Constant score	43.75 ± 18.18	74.08 ± 8.35	0.0001

**Table 5:** SSV: comparison between and within groups.

SSV	One Year (Mean ± SD)	Last Follow-Up (Mean ± SD)	*p*-Value
Group A	72.50 ± 12.25	72.50 ± 13.89	1
Group B	81.67 ± 9.85	82.50 ± 8.86	0.952
*p*-value	0.080	0.101	

SSV: shoulder subjective value.

**Table 6:** Constant score: comparison between and within groups.

Constant Score	One Year (Mean ± SD)	Last Follow-Up (Mean ± SD)	*p*-Value
Group A	68.50 ± 12.29	66.85 ± 15.99	0.616
Group B	72.38 ± 8.03	74.08 ± 8.35	0.684
*p*-value	0.467	0.134	

**Table 7:** Comparison between patients who underwent DAIR and patients who directly underwent the two-stage revision.

Outcome	DAIR (*Mean* ± *SD*)	No DAIR (*Mean* ± *SD*)	*p*-Value
N pts	3	13	
SSV	42.5 ± 45.96	63.13 ± 18.11	0.298
Constant score	55.67 ± 27.30	65.60 ± 16.85	0.448

## Data Availability

The data presented in this study are available on request from the corresponding author.
